# Foundations in Racism: a Novel and Contemporary Curriculum for Child and Adolescent Psychiatry Fellows

**DOI:** 10.1007/s40596-021-01396-0

**Published:** 2021-02-11

**Authors:** Rupinder K. Legha, Misty Richards, Sheryl H. Kataoka

**Affiliations:** David Geffen School of Medicine at University of California Los Angeles (UCLA), Los Angeles, CA USA

Studies have documented the far-reaching health consequences of racism for over two decades [1]. Despite this, the medical profession has avoided identifying racism as a root cause of health inequities [2]. Within psychiatric education, racism has primarily been conceptualized as part of cultural competency and the social determinants of health, with implicit bias as a more recent focal point [3]. However, in a recent study, child and adolescent (CAP) fellowship directors reported that structural and historical social determinants (e.g., “institutionalized poverty and racism, community history”) are taught less effectively than all other social determinant categories [4]. This educational gap exists, despite growing evidence that systemic racism contributes to the disproportionality of Black compared to white youth in child welfare, juvenile justice, and special education systems [5].

The deaths of Trayvon Martin, George Floyd, Breonna Taylor, and countless other individuals due to police brutality have triggered critiques of the silence on racism in medical training. Faculty and students have insisted on integrating structural competency and antiracist pedagogy into medical education, so the next generation of physicians can eliminate racism from clinical practice and challenge societal injustice more broadly [6]. Furthermore, the history of white supremacy in America is increasingly considered requisite learning for diagnosing and combatting structural racism, which sustained equity requires [7, 8].

To our knowledge, however, few published models about racism in residency education exist. A search for “racism” in the Association of American Medical College’s medical education database (MedEdPORTAL) reveals six curricula involving race or racism, but only four explicitly state those terms in their titles and are directed towards medical trainees. Only one curriculum is for psychiatry trainees, from the Massachusetts General Hospital/McLean Psychiatry residency program, comprised of four 50-min interactive sessions on addressing racial inequities [9]. None is for child psychiatry fellows. Furthermore, few scientific publications have described curricula for psychiatry trainees that focus on the historical roots of racism and how to develop and implement such curricular materials [9].

This educational case report strives to address this gap by describing the University of California Los Angeles (UCLA) CAP Fellowship Program’s novel *Foundations in Racism* curriculum, first piloted in 2017 to first-year child psychiatry fellows. The curriculum emphasizes how the American Indian Genocide and slavery shaped racism’s many forms, drive (mental) health inequities, and influence the practice of psychiatry. Beyond highlighting the curricular content, we emphasize our process, providing a roadmap for other psychiatry training programs aiming to develop and deliver similar curricula.

## Preimplementation Considerations

### Racial Climate Assessment

Because racism is an important yet emotionally provocative topic that is inadequately discussed or taught [6], one consideration prior to implementing a racism curriculum is assessing institutional racial climate. Formal assessments relying, for example, on organizational climate surveys, or more informal considerations can help gauge readiness and receptivity of the environment for this content [10]. We took an informal approach by examining the existing CAP fellowship curriculum in addition to tracking the diversity of trainees and faculty over the past 10 years.

The November 2017 tragedies surrounding the Unite the Right Rally in Charlottesville, Virginia, amplified the lack of discourse around racism within our CAP division and fellowship, provoking a self-assessment of the fellowship’s didactic content related to discrimination and racism. Among the 170 lectures, we identified three related to culture and disparities, two pertaining to LGBTQ and gender non-conforming youth, and none explicitly focused on racism. As a result, the fellowship program directors (the second and senior authors) invited the lead author, a second-year fellow at the time, to develop de novo content based on her expertise teaching about racism and health equity.

We also examined our trainees’ demographics as we prepared to implement holistic review principles into our recruitment and selection process. The CAP fellowship had a history of recruiting 0–2 underrepresented minority (URM) fellows out of 7 per year, averaging .9 URM fellows annually over the previous 10 years. We also noted that our CAP division and larger departmental leadership was primarily white and male.

We concluded that the training program would benefit from piloting this curriculum and ongoing development of holistic review for fellowship recruitment. However, we also acknowledged that our division and department needed additional (structural) reforms including but not limited to faculty training in racism and antiracism, as well as improved recruitment, retention, and promotion of URM faculty. Finally, we recognized the challenges facing a woman of color piloting this novel curriculum as a trainee, in this white-male-dominant environment, with limited URM trainee presence, and no prior didactic content about racism.

### Structure and Process

The overarching goal of this curriculum is to provoke meaningful racial dialogue and to enhance racial consciousness, with the long-term objective of improving clinical care. We prioritized teaching it to the first-year fellows early in training in order to frame later discussions of health inequities and child systems of care in the context of racism. We also intentionally delivered this content 2 months into the academic year allowing the fellows to establish some group cohesion during their weekly, year-long seminar. Finally, we taught this curriculum in a small class of 12 fellows (7 from the primary program and 5 from affiliated sites) to promote dialogue.

## Curriculum Implementation

At least one of the fellowship program directors (the second and senior authors) attended to observe and offer senior support to the second-year fellow (lead author) delivering the curriculum as part of her elective. Each of the four 1-h sessions was a facilitated, interactive discussion. The original learning objectives of this curriculum were to (1) understand how racism shapes the manifestation, diagnosis, and treatment of mental illness; (2) recognize historical forces as foundational for making sense of racial inequities; (3) improve awareness about race and racism; and (4) recognize the role of policies and systems in maintaining racism.

Conceptual frameworks derived from several key documents, including The National Academy of Medicine’s *Framing the Dialogue on Racial Equity*, which emphasizes the importance of historical contexts and white dominant narratives in shaping interpretation of information [11]. Derald Sue’s work on racial microaggressions and Camara Jones’ multi-level model of racism provided focal points for defining racism [12, 13]. Ibram X Kendi’s *Stamped from the Beginning* and Ta-Nehisi Coates’ “The Case for Reparations” underscored the centrality of racist policy in shaping inequity [14, 15].

We framed biomedical content with contemporary works from history and journalism to bridge the disconnection between clinical work and the racially charged events shaping our country. Photographs, documentary footage, and Ted Talks were used to illustrate racism’s clinical toll, to challenge prevailing narratives pertaining to democracy and justice in this country, and to guard against solely intellectualizing racism. The Equal Justice Initiative and Southern Poverty Law Center’s Teaching Tolerance websites provided accessible historical content [16, 17]. The progression of lectures was intentional: the first two lectures centered on theory and the scientific literature and were less emotionally provocative than the latter two lectures on trauma, human atrocity, and injustice. See Table 1 for details of sessions and Table 2 for session strategies.

**Table 1:** Overview of four lectures

	Lecture 1: racial Discrimination and Health	Lecture 2: Let’s talk about racism: multi-level framework	Lecture 3: historical trauma—Pine Ridge Reservation case study	Lecture 4: the legacy of slavery in American Medicine and Psychiatry
Content	-Review of racial discrimination literature and the primary dialogue about racism in the biomedical literature -Emphasize physiological mechanisms of racial discrimination	-Five-level social-ecological model of racism (internalized, interpersonal, institutional/structural, ideological, and policy) -Definitions, mechanisms, and key examples for each level	-The Pine Ridge Reservation’s health inequities (substance use, suicide, and mortality) as they pertain to the American Indian genocide -Historical trauma concept -The Holocaust literature’s emphasis on trauma’s biological and psychological harm	-Slavery as the DNA for all major American institutions -American medicine and psychiatry’s complicity with slavery through (1) professional exclusion of Black physicians, (2) segregation and denial of care, (3) research abuses (beyond Tuskegee), (4) silence about injustice and inequity, and (5) narrative of biological racial difference
Primary learning objective	Understand how racism shapes the manifestation, diagnosis, and treatment of mental illness a. Identify the key biomedical literature related to racism, as well as its limitations b. Identify role of racism in clinical care	Improve awareness about race and racism a. How to identify and name racism b. Identifying racist policies rather than individual racist behavior, in shaping inequities	Recognize history as a foundation for making sense of racial inequities a. How historical atrocities such as the American Indian Genocide and slavery are critical to understanding trauma b. How historical racism is associated with clinical care and health care more broadly	Recognize role of policies and systems in maintaining racism Emphasizing that contemporary health disparities affecting Black Americans originated during slavery and have evolved
Required readings	Priest N. et al. A systematic review of studies examining the relationship between reported racism and health and wellbeing for children and young people. Social Sci Med. 2013;95:115–27	Jones C. Levels of racism: a theoretic framework and a gardener’s tale. Am J Pub Health. 2000;90(8):1212–5	Heart MY & Debruyn LM. The American Indian Holocaust: Healing historical unresolved grief. Am Indian Alsk Native Men Health Res. 1998;8(2):56–78	Gamble VN. Under the shadow of Tuskegee: African Americans and health care. Am J Pub Health. 1997; 87(11):1773–8
Other key references	Mohatt NV & Tebes K. Historical trauma as public narrative: a conceptual overview of how history impacts present day health. Soc Sci Med. 2014; 106:128–36	Kendi IX. Stamped from the beginning: the definitive history of racist ideas in America. New York: Nation Books; 2016	Sotero, M. A conceptual model of historical trauma: implications for public health practice and research. J Health Disp Res Prac. 2006; 1:93–108	Gordon-Achebe K et al. Origins of racism in American medicine and psychiatry. In: Medlock M, Shtasel D, Trinh N-H, Williams DR, editors. Racism and psychiatry: contemporary issues and interventions. Champagne: Humana Press; 2019. pp. 3–19
Journalism/media (videos, artwork)	Bryan Stevenson TED Talk, “The Opposite of Poverty is Justice.” David R. Williams TED Talk, “How Racism Makes Us Sick.”	Allen D. Life of a South Central statistic. New Yorker Magazine. 2017; July 24.	Aaron Huey TED Talk, “American Prisoners of War.”	Equal Justice Initiative Video, #Slavery Evolved. Governor Ralph Northam’s blackface photos.

**Table 2:** Goals, objectives, and strategies

Overarching curriculum components	Strategy
Bridge disconnect between clinical work and contemporary racially charged events.	Draw on contemporary journalism and other media, such as documentaries, Ted Talks, and podcasts; draw upon non-biomedical resources from the humanities and advocacy groups (e.g., Equal Justice Initiative).
Engage in meaningful racial dialogue.	Create space of safety through choice of lecture space and size, lecturer training, timing of lectures, presence of leadership, and lecturer offering individual discussion outside of lecture sessions.
Foster self-reflection and racial consciousness building.	Lecturer models, drawing upon an intersecting axes of privilege, domination, and privilege framework and admitting to her own racism.
Prevent deepening of participants’ racial bias.	Participants encouraged to share their own experiences and beliefs about racism. Race defined as a social and political construct, not a biological one. Honest and explicit language, e.g., racism and white supremacy rather than diversity and inclusion.
Specific learning objectives	Strategy
Understand how racism can shape the manifestation, diagnosis, and treatment of mental illness.	Introduce the primary biomedical literature about racial discrimination and health, emphasizing key findings and limitations. Provide explicit definitions of racism using a multi-level model of framework.
Recognize history as a foundation for making sense of racial inequities.	Draw upon award-winning books and authors from the humanities and advocacy resources, such as the Equal Justice Initiative website; emphasize the American Indian genocide and slavery as defining events shaping health disparities in America.
Improve awareness about race and racism.	Provide an evidence-based framework for understanding racism, making visible the racism embedded in American medicine and psychiatry. Describe how these frameworks shape diagnosis and treatment.
Recognize role of policies and systems in maintaining racism.	Shift the focus away from individual intentions and behaviors by instead emphasizing racist policies’ (e.g., The War on Drugs and Jim Crow lynching and terrorism) pivotal role in creating inequity.
Challenges	Strategy/next steps
Participant disengagement and discomfort.	Require that participants put laptops and cell phones away to ensure respect for the gravity of the content. Set the frame and create a space of safety. Teach participants about white fragility and racial defense mechanisms.
Content can be traumatic for URM trainees, amplified by faculty’s lack of knowledge.	Corresponding faculty development. Lecturer should be available to provide supervision and support for URM trainees. Trainees should be allowed to opt out of any traumatizing content or discussions.
Four lectures are limited in their ability to yield more racially equitable clinical practices.	Be forthcoming about the limitations of the content. Emphasize the impactful role of institutional racism. Forthcoming curricular content will articulate antiracist clinical practices.
The lecture content deals broadly with racism and is not specialized enough for child and adolescent psychiatry fellows.	Consider the four lectures a pilot for introducing basic concepts pertaining to racism and white supremacy. Use feedback from pilot effort to innovate new content that is more specialized for child and adolescent psychiatry fellows’ stage of learning.

The curriculum began by drawing upon a critical race theory approach to create a space for self-reflection and racial consciousness. The lecturer analyzed her own racial identity as a South Asian woman, utilizing an intersecting axis of privilege, domination, and privilege framework [18]. She admitted that her own racism would inevitably manifest in the lecture content and that having the platform to discuss this topic was in and of itself a manifestation of her racial privilege. Participants were then invited to discuss how these intersecting axes shape their own experiences and beliefs about racism. To foster discussion and emphasize ongoing learning, she foreshadowed periodically soliciting questions, impressions, and emotional reactions and offered to continue discussions after class as needed. To help protect against the content deepening racial bias, a potential risk of such curricula, race, was explicitly defined as a social and political construct, rather than a biological one. The lecturer also acknowledged how the racial terms she would be using—such as “white” and “Black”—could be racist if used without humility or to promote a narrative of biological racial difference [19].

The stages of racial consciousness and identity development may differ significantly for trainees. This content can provoke a range of emotions, including sadness, guilt, defensiveness, and even hopelessness, particularly among white trainees [12]. To support trainees effectively, the lecturer was mindful of, though not presumptuous about, trainees’ personal experiences of racism and degree of racial consciousness. We believe that we further minimized harm by setting an appropriate frame at the beginning and providing support for continued processing of content individually.

## Preliminary Evaluation

A brief, anonymous questionnaire was given pre and post curriculum, as part of a program quality improvement effort. Of the 12 fellows attending this curriculum, 11 completed the pretest and 12 completed the post-test. Only 18% of fellows had previously received racism education in clinical training at baseline. When asked if they understood how to define types of racism, 64% at pre- and 83% at post-test agreed. When asked if they felt confident in their knowledge about how racial discrimination affects mental and physical health, 73% at pre- and 83% at post-test agreed. Interestingly when asked about whether they consider the role of racism in the lives of patients, fewer fellows agreed at post- (83%) than pretest (91%). Finally, when asked about feeling confident in their ability to evaluate the role of racism in patients’ lives, 36% at pre- and 58% at post-test agreed. Given this pilot data’s small sample size, it is unclear whether these percentages are statistically significant.

Qualitative comments at post-test were unanimously positive but suggested that continued dialogue about racism throughout training is needed (i.e., “This is such an important topic but I still feel so ill equipped to apply it in clinical practice.”) Another fellow commented: “this was the best lecture series and we need more…” and another suggested that broader education on racism should be threaded throughout education: “This should be a college general education class for mass distribution!”

Anecdotally, URM fellows expressed gratitude for seeing material pertaining to racism in medicine and psychiatry delivered in an honest, substantive, and historically informed fashion, often for the first time. Several fellows sought individual mentorship and support from the lecturer outside of class to discuss the racism they face in training. Other attendees reported subsequently having meaningful conversations about racism for the first time in their professional space. Several trainees requested that their teaching faculty receive the same didactic content.

In contrast, some participants were visibly disengaged and on their laptops or phones, which may have been an emotional defense mechanism. Although participants were given the opportunity to step out of the room if any content was upsetting or triggering, no one left the room or demonstrated visible indicators of discomfort with the content. Though the baseline survey suggested that most participants felt confident about their knowledge of racism and health, the questions and dialogue generated by the sessions suggested that many of the concepts were new.

## Challenges and Next Steps

To our knowledge, this curriculum is the first to provide a four-session exploration of the history of racism in medicine, psychiatry, and society for CAP fellows. Camara Jones’ schema of racism as a multi-level entity and history as a foundation for understanding racism and associated disparities are core components of other racial equity materials and have been a practical starting point for integrating racism into psychiatry didactics [9, 13]. However, our reliance on media and content from the humanities, journalism, and advocacy groups is a novel approach, and we believe it enhanced the emotional resonance of the content.

This curriculum’s broad focus on racism has facilitated its adaptation for medical students, medical school faculty, psychology interns, and general psychiatry residents. Based on feedback, we are developing additional educational material about how to engage in antiracist clinical care and reparations for slavery as a public health intervention [20, 21]. This next phase of curriculum content, entitled Antiracism in Medicine and Psychiatry, will feature Ibram X Kendi’s *How to Be An Antiracist* [22]. We believe the first four lectures, which focus on defining and elaborating on racism’s role in medicine, psychiatry, and beyond, provide the foundation for the next phase of learning related to meaningful antiracist action and clinical care. As the curriculum’s audience expands to psychiatry residents and medical students, we plan to develop more specialized lectures for child and adolescent psychiatry fellows. School pushout (the practice of using punitive disciplinary practices to exclude students of color from class and push them out of school settings, increasing their risk of juvenile justice system involvement), how children develop bias, and how to discuss racism in a developmentally appropriate fashion are potential topics.

This curriculum has several limitations. Though course evaluations were positive, the original learning objectives, particularly improving clinical care, were ambitious for four lectures. Creating this curriculum de novo in the absence of best practices for teaching child psychiatry fellows about racism made pinpointing and achieving these objectives challenging. However, we approached this pilot as a quality improvement effort, believe the curricular development centered on antiracism will advance the original learning objectives, and plan to expand content beyond the four original lectures.

We also plan to conduct a more rigorous mixed-methods evaluation of this curriculum. Ideally, future evaluations would be conducted independently of the lecturer and assess for trainees’ knowledge, attitudes, awareness, beliefs, and defense mechanisms about racism in more depth. Qualitative key informant interviews could yield additional information about how the curriculum is being used and “digested” by participants and if there are potentially negative effects (i.e., exacerbation of racial bias). This more rigorous evaluation could enhance defining and achieving learning objectives while innovating additional curricular content, which is certainly our goal, given racism’s growing recognition as a public health crisis of global concern [8]. Despite its limitations, our pilot’s content and process can guide other psychiatry training programs’ antiracist curricular efforts, which may be heightened during this moment of profound historical reckoning. As these efforts unfold, we recommend evaluating educators’ experiences developing and teaching this crucial content, given its complexity and the expertise needed to facilitate dialogue and process emotional reactions [12].
